# LpxR and PagL expression in live attenuated auxotrophic *Pseudomonas aeruginosa* vaccines modulates lipid A reactogenicity *in vitro* while preserving immunogenicity

**DOI:** 10.3389/fcimb.2025.1664169

**Published:** 2025-10-16

**Authors:** Miriam Moscoso, Víctor Fuentes-Valverde, Patricia García, Juan A. Vallejo, Jesús Pérez-Ortega, Rebeca Santamarina-Fernández, Ana Candela, Marina Oviaño, Jesús Arenas, Germán Bou

**Affiliations:** ^1^ Servicio de Microbiología, Instituto de Investigación Biomédica de A Coruña (INIBIC), Complexo Hospitalario Universitario de A Coruña (CHUAC), Sergas, A Coruña, Spain; ^2^ Centro de Investigación Biomédica en Red de Enfermedades Infecciosas (CIBERINFEC), Instituto de Salud Carlos III, Madrid, Spain; ^3^ Department of Molecular Microbiology, Faculty of Science and Institute of Biomembranes. Utrecht University, Utrecht, Netherlands; ^4^ Unit of Microbiology and Immunology, Faculty of Veterinary, University of Zaragoza, Agroalimentary Institute of Aragon, IA2. II2, Zaragoza, Spain; ^5^ Departamento de Fisioterapia, Medicina y Ciencias Biomédicas, Universidad de A Coruña, A Coruña, Spain

**Keywords:** *Pseudomonas aeruginosa*, lipopolysaccharide, lipid A modifying-enzymes, live vaccines, D-glutamate auxotrophy, humoral immunity, protective efficacy, reactogenicity

## Abstract

**Introduction:**

*Pseudomonas aeruginosa* is a Gram-negative opportunistic pathogen responsible for severe respiratory tract infections. We previously developed a live attenuated auxotrophic vaccine candidate, PAO1 Δ*murI*, which conferred protection in murine infection models but exhibited significant reactogenicity when administered intranasally. To reduce the toxicity of PAO1 Δ*murI* without compromising its protective efficacy, we engineered strains with a modified lipid A structure, as lipid A is one of the main toxic components of whole-cell vaccines.

**Methods:**

Two lipid A-modifying enzymes, LpxR and PagL were overproduced in PAO1 Δ*murI* derivatives. The resulting lipopolysaccharide (LPS) was analyzed by MALDI-TOF mass spectrometry. *In vitro* assays with HEK293-Blue reporter cells expressing murine and human Toll-like receptor 4 (TLR4) were used to assess LPS-associated toxicity, while *in vivo* reactogenicity and protective efficacy were evaluated in a murine acute pneumonia model.

**Results:**

LPS extracted from the wild-type strain showed heterogeneous lipid A structures with varying degrees of acylation, and a predominant penta-acylated species. Expression of LpxR led to enrichment in tetra-acylated species, while PagL expression reduced the heterogeneity observed in the wild type. Both mutant strains showed decreased TLR4 activation *in vitro* as compared to the wild type. In mice, lipid A-modified derivatives retained protective efficacy; however, no reduction in reactogenicity was observed.

**Discussion:**

Lipid A modifications mediated by LpxR and PagL attenuated TLR4 signaling *in vitro* but were insufficient to reduce *in vivo* reactogenicity. Additional modifications or targeting of other toxic components may be required. This strategy may serve as an initial basis for optimizing live attenuated *P. aeruginosa* vaccines, although additional approaches will likely be necessary to achieve substantial improvements.

## 1 Introduction

*Pseudomonas aeruginosa* is an opportunistic pathogen responsible for a wide range of infections, particularly in immunocompromised individuals or those with skin or mucosal lesions (Killough et al., 2022). In 2019, this pathogen was ranked among the top five bacterial pathogens associated with global mortality, mainly due to lower respiratory tract and bloodstream infections (Collaborators, G. B. D. A. R, 2022). In addition, due to its notable resistance to antimicrobials (Mulani et al., 2019; De Oliveira et al., 2020), the World Health Organization has classified carbapenem-resistant *P. aeruginosa* as a “high priority” (Priority 2) pathogen (Jesudason, 2024).

Given the limited effectiveness of antibiotics against multidrug-resistant *P. aeruginosa* and the slow development of new antimicrobials, vaccines represent a promising preventive strategy, particularly for high-risk individuals (Killough et al., 2022). Various vaccine formulations have been proposed, yet only a limited number have advanced to clinical trials, and none have received regulatory approval to date (Santamarina-Fernandez et al., 2025). We previously evaluated an attenuated D-glutamate auxotrophic, PAO1 Δ*murI*, which proved safe and effective in a murine sepsis model (Cabral et al., 2017). Intranasal (IN) administration of this strain elicited systemic and mucosal antibodies, activated IL-17-producing CD4^+^ T cells, and significantly improved survival rates in mice with acute pulmonary infections caused by cytotoxin-producing *P. aeruginosa* strains (Cabral et al., 2017, 2020). However, high-dose IN vaccination was associated with adverse effects, likely due to lipopolysaccharide (LPS) endotoxin activity (Cabral et al., 2020; Fuentes-Valverde et al., 2022).

One of the most relevant virulence factors in *P. aeruginosa* respiratory infections is the LPS, the primary structural component of the outer membrane in Gram-negative bacteria. LPS consist of three main regions: lipid A, the oligosaccharide core, and the O-antigen. It acts as a physical barrier, stimulates the production of reactive oxygen species, and triggers pulmonary inflammation. Additionally, LPS contributes to antibiotic resistance and tissue damage through the endotoxin activity of lipid A (Huszczynski et al., 2020). The canonical lipid A is composed of a β(1’,6)-linked glucosamine disaccharide, with phosphate groups at the 1 and 4’ positions, fatty acids at positions 2 and 2’ linked via amide bonds, and fatty acids at positions 3 and 3’ linked via ester bonds. Secondary acyl chains can also be linked to the primary ones. However, the number, length and pattern of acyl chains, as well as additional chemical modifications, vary among bacterial species. Some strains can further modify the lipid A structure in response to environmental cues producing lipid A-modifying enzymes (Simpson and Trent, 2019).

**Figure 1:**
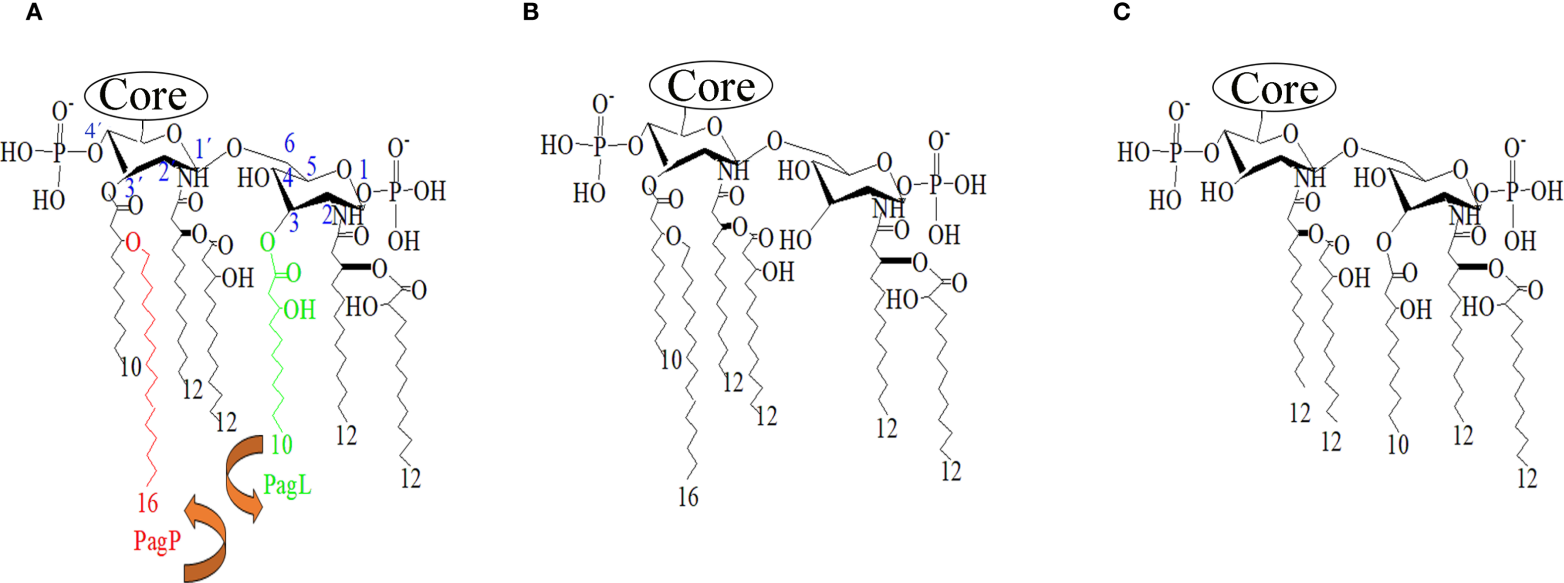
Lipid A structures of *P. aeruginosa.* **(A)** Typical lipid A produced by *P. aeruginosa* strain PAO1. Acyl chains targeted by the deacylases PagL (green) and the acyltransferase PagP (red). The position of the residues on the glucosamine backbone is indicated in blue. **(B)** Predicted structure after overexpression of PagL. **(C)** Predicted structure after overproduction of *S.* Typhimurium LpxR. Structures were generated using ChemDraw software.

In *P. aeruginosa*, lipid A exhibits as a heterogeneous mixture of penta- to hepta-acylated species. Environmental isolates or laboratory-adapted strains are enriched in penta-acylated bis-phosphorylated species, which consist of a diglucosamine backbone with one 3-hydroxydecanoic acid (3OH-C10) and two 3-hydroxydodecanoic acid (3OH-C12) as primary fatty acids, and secondary acyl chains such as 2OH-C12 and dodecanoic acid (C12) chain and two phosphate residues (**Figure 1A**). In contrast, clinical isolates from cystic fibrosis patients often synthesize a hexa-acylated lipid A due to the addition of a palmitate chain mediated by the outer membrane enzyme PagP (Ernst et al., 1999). A less frequent hepta-acylated lipid A variant has also been identified in cystic fibrosis patients, which retains 3OH-C10 at position 3, presumably due to the loss of activity of the acyltransferase PagL (Cigana et al., 2009; Geurtsen et al., 2005). PagL is known to hydrolyze the ester bond at position 3 of lipid A (**Figure 1B**). This modification of lipid A is associated with increased resistance to β-lactam and polymyxin B antibiotics, though not to aminoglycosides, and reduced recognition by the Toll-like Receptor 4 (TLR4) (Ernst et al., 2006; Kawasaki et al., 2004; Ernst et al., 2007; Zhang et al., 2017). Other modifications have also been identified in *P. aeruginosa* isolates from cystic fibrosis patients, such as alterations to the phosphate groups through the incorporation of 4-amino-4-deoxy-L-arabinopyranose (Ara4N) by ArnT or phosphoethanolamine (pEtN) at positions 1 and 4’ by EptA (Moskowitz et al., 2004; Nowicki et al., 2015; Simpson and Trent, 2019). In contrast, *P. aeruginosa* lacks LpxR, a 3’-O-deacylase identified in *Salmonella* Typhimurium that modulate lipid A structure by removing the primary acyl chain at the 3’ position, thereby reducing activation of the human TLR4/MD2 complex (Reynolds et al., 2006) (**Figure 1C**).

LPS activates the immune system by triggering the production of proinflammatory interleukins. Once released from the bacterial surface/membrane, LPS monomers are transferred to the TLR4/MD-2 complex on immune cell surfaces through two accessory proteins: LPS-binding protein and CD14. Recognition by TLR4/MD-2 initiates an intracellular signaling cascade via either MyD88 (myeloid differentiation primary response 88) or TRIF (TIR-domain-containing adaptor-inducing interferon-β). The MyD88-dependent pathway induces pro-inflammatory cytokines, whereas the TRIF-dependent pathway promotes interferon-regulated cytokines with weaker inflammatory activity (Simpson and Trent, 2019). The lipid A structure strongly influences TLR4 activation. Particularly, the number of acyl chains is a critical determinant (Needham et al., 2013). Hexa-acylated lipid A is the most potent agonist of human TLR4 (hTLR4), whereas penta-acylated form are approximately 100 times less active, and the tetra-acylated forms, act as antagonist (Park et al., 2009).

Modulating the expression of lipid A biosynthesis or modifying enzymes by genetic engineering has been successfully used to reduce the toxicity of whole-cell *Bordetella* vaccines in humans and animals (Arenas et al., 2020; Perez-Ortega et al., 2021). In this study, we investigated whether increasing the production of antagonist or low-activity lipid A species in the *P. aeruginosa* PAO1 Δ*murI*, through heterologous expression of LpxR and PagL, could attenuate LPS-mediated reactogenicity by reducing TLR4-driven inflammatory signaling.

## 2 Materials and methods

### 2.1 Bacterial strains, growth conditions and plasmids

The bacterial strains and plasmids used in this study are showed in **Table 1**. All strains were cultured in Luria-Bertani (LB) broth (10 g/L tryptone, 5 g/L yeast extract and 10 g/L NaCl) with aeration at 37°C or on LB plates with 2% agar. For plasmid selection in *Escherichia coli*, apramycin (Sigma-Aldrich, Inc.) or ampicillin (Sigma-Aldrich, Inc.) were added at a concentration of 50 and 100 µg/ml, respectively. *P. aeruginosa* PAO1 Δ*murI* was cultivated in LB supplemented with 10 mM D-glutamate (Sigma-Aldrich, Inc.). For gene expression and plasmid maintenance in PAO1 Δ*murI*, 1 mM isopropyl-β-D-1-thiogalactopyranoside (IPTG; Thermo Scientific) and apramycin at 200 µg/ml were added to the medium supplemented with 8 mM D-glutamate.

**Table 1:** Bacterial strains and plasmids used in this study.

Strain or plasmid	Relevant features	Reference
Strains		
*P. aeruginosa*		
PAO1	Reference strain. Isolate from a patient with a burn wound infection.	CECT 4122; (Stover et al., 2000)
PAO1 Δ*murI*	PAO1 derivative, ΔPA4662. Auxotroph for D-glutamate.	(Cabral et al., 2017)
PA14	Hypervirulent strain isolated from a patient with a burn wound infection.	(Lee et al., 2006)
PAO1 Δ*murI* p*lpxR*_ST_	PAO1 Δ*murI* derivative with the plasmid pMMB67EH-LpxR_ST_, which overexpresses the deacylase LpxR from *Salmonella* Typhimurium. D-glutamate auxotroph. Apr^R^.	This study
PAO1 Δ*murI* p*pagL*	PAO1 Δ*murI* derivative with the plasmid pMMB67EH-PagL_Pa_, which overexpresses the deacylase PagL from *P. aeruginosa*. D-glutamate auxotroph. Apr^R^.	This study
*E. coli and Salmonella*		
DH5α	*ΔlacZΔM15 Δ*(*lacZYA-argF*)*U169 recA1 endA1 hsdR17*(*rK^-^mK^+^*) *supE44 thi-1 gyrA96 relA1*	Lab collection (Hanahan, 1983)
*Salmonella enterica* subsp *enterica* LT2	Serovar Typhimurium, O:4,[5],12:H:i:1,2	(McClelland et al., 2001)
Plasmids		
pIJ773	pBluescript II SK(+) derivative containing the cassette *aac(3)-IV* – *oriT* (RK2) and flanking FRT sites; Apr^R^, Amp^R^	(Gust et al., 2003; Huang et al., 2014)
pMMB67EH-PagL_Pa_	pMMB67EH derivative harboring the *pagL* gene from *P. aeruginosa* cloned under the inducible *tac* promoter. Amp^R^.	(Geurtsen et al., 2006)
pMMB67EH-PagL_Pa__Apra	pMMB67EH-PagL_Pa_ derivative containing the *aac(3)-IV* gen of pIJ773. Apr^R^.	This study
pMMB67EH-LpxR_ST__Apra	pMMB67EH derivative harboring *lpxR* from *S.* Typhimurium cloned under the inducible *tac* promoter and the *aac(3)-IV* gen of pIJ773. Apr^R^.	This study

Amp^R^, ampicillin-resistant; Apr^R^, apramycin-resistant; CECT, Spanish Type Culture Collection.

### 2.2 DNA manipulation

All primers used in this study are listed in **Table 2**. Plasmid DNA from *E. coli* was obtained with the High Pure Plasmid Isolation Kit (Roche Diagnostics, S.L.). PCR amplifications were performed using Expand High Fidelity PCR System (Roche Diagnostics, S.L.) and GoTaq Flexi DNA polymerase (Promega Biotech Ibérica, S.L.). The restriction enzymes FastDigest^®^, FastAP Alkaline Phosphatase (Thermo-Fisher Scientific) and T4 DNA ligase (Promega Biotech Ibérica, S.L.) were used as recommended by the supplier. To construct plasmid pMMB67EH-PagL_Pa__Apra, an apramycin resistance cassette was amplified from plasmid pIJ773 using primers Apra Cas PuvI F and Apra Cas PuvI R, digested with restriction enzymes, and cloned into plasmid pMMB67EH-PagL_Pa_. The resulting plasmid derivative was called pMMB67EH-PagL_Pa__Apra. For the generation of pMMB67EH-LpxR_Apra plasmid, the *lpxR* gene was amplified from *S.* Typhimurium LT2 genomic DNA, digested with appropriated restriction enzymes, and cloned into plasmid pMMB67EH-PagL_Pa_. Then, the apramycin resistance cassette was inserted as described above. Both plasmid constructs were verified by Sanger sequencing using primers pMMB F and pMMB R, which anneal to the flanking regions of the inserted gene.

**Table 2:** List of primers used in this study.

Primers	Sequence	UPL probe
PCR, sequencing and/or cloning		
pMMB F	AATCATCGGCTCGTATAATGT	_
pMMB R	CCTGGCAGTTCCCTACTCT	_
LpxR Fw-NdeI	GCGCGC**CATATG**AACAAA TACAGCTATTG	_
LpxR Rev-BamHI	GCGCGC**GGATCC**TCAGAAAA AGAACGTTATAT	_
Apra Cas PuvI F	**CGATCG**ATCGATTCCGGGGA TCCGTCGACC	_
Apra Cas PuvI R	**CGATCG**ATCGTGTAGGCTGGAG CTGCTTC	_
RT-qPCR		
*eptA*#156_left	GCCGGTACCACCTACTTCAT	#156
*eptA*#156_right	GGTTGGTCTGCATGACGTTG	#156
*pagL*#124_left	CCTGAACTTCGAAGACCGCA	#124
*pagL*#124_right	AATAGTGGATCGCCCGAACG	#124
*pagP*#26_left	ATCTCATCCTCAGCCTGCTG	#26
*pagP*#26_right	CTGCAGGTACCAGAAGTCGC	#26
*lpxRst*#124_left	ATTCTCCATCAGGCGCCAAT	#124
*lpxRst*#124_right	TCCGGTATGAAGAAAGGCGC	#124
*rpoS*#63_left	AAGAAGGGCCGGAGTTTGAC	#63
*rpoS*#63_right	ACGACTCGTCCAGCATGATG	#63
apra#96_left	CCATCCATTTGCCTTTGCGG	#96
apra#96_right	GCAGGGGCAATGGATCAGAG	#96

F, forward primer; R, reverse primer. *Restriction sites introduced into the sequence for cloning are indicated in bold. UPL, TaqMan probes from the Universal Probe Library (Roche, Germany).

### 2.3 Transformation by electroporation

To prepare electrocompetent cells of *E. coli* or *P. aeruginosa*, a 1:100 dilution of an overnight culture was inoculated into fresh LB medium with the necessary supplements. The culture was incubated at 37°C with continuous shaking until an optical density at 600 nm (OD_600_) of 0.7 was reached. The bacterial cells were harvested by centrifugation (3,900 × *g*, 20 min, 4°C), washed three times with cold 10% glycerol, and gradually concentrated in the same solution. The final suspension, concentrated 130-fold, was aliquoted into volumes of 50-100 μL, which were used immediately or stored at –80°C for later use (Lofblom et al., 2007). Electroporation was performed following the procedure described by Dower et al. (1988) (Dower et al., 1988), with slight modifications. For *E. coli*, 50 μL of electrocompetent cells were transformed with 2-4 μL (approximately 1 μg) of plasmid DNA obtained from a minipreparation, which had been microdialyzed to remove residual salts. The mixture of competent cells and DNA was incubated on ice for 5 min and then transferred to a 0.1 cm gap electroporation cuvette (Bio-Rad). A Gene Pulser Xcell system (Bio-Rad, USA) was used for electroporation with the following parameters: 25 μF, 200 Ω, and 1,800 V. For *P. aeruginosa*, the procedure was similar, but used a larger volume of electrocompetent cells (200 μL) mixed with the plasmid DNA in a 0.2 cm gap cuvette (Bio-Rad), and a voltage of 2,500 V was applied. In both cases, immediately after the pulse, 1 mL of SOC medium (0.5% yeast extract, 2% tryptone, 10 mM NaCl, 2.5 mM KCl, 10 mM MgCl_2_, 10 mM MgSO_4_, 20 mM glucose) supplemented as required was added, and the cell suspension was incubated at 37°C with gentle shaking for 1 hour. After incubation, potential transformants were plated on LB agar supplemented with 8 mM D-glutamate and the appropriate selection antibiotic.

### 2.4 Growth and viability curves

To generate growth and viability curves for *P. aeruginosa* PAO1, D-glutamate auxotroph PAO1 Δ*murI* and its derivatives expressing enzymatically modified LPS (namely, PAO1 Δ*murI* p*lpxR*_ST_ and PAO1 Δ*murI* p*pagL*), a single colony of each strain was inoculated into 5 mL of LB medium supplemented with either 8 mM D-glutamate and 200 μg/mL apramycin (LGA medium), when were required. Cultures were incubated with shaking at 37°C for 16 hours. Subsequently, a 1:50 dilution was made into 100 mL of fresh medium, and 1 mM IPTG was added to induce gene expression. These cultures were then incubated at 37°C with shaking (180 rpm) for 8 hours. Samples were taken every hour to measure culture turbidity by determining the OD_600_ with a spectrophotometer. To assess viability, the drop-plating technique was used (Sieuwerts et al., 2008). Briefly, 1:10 serial dilutions of the different aliquots were prepared, and 5 μL of each dilution was plated in triplicate on supplemented solid medium. Plates were incubated at 37°C for 24 hours, and spots with a colony forming units (CFU) count of 30 or more were recorded.

### 2.5 Lipid A extraction and structural analysis

Lipid A was extracted using the MBT Lipid Xtract Kit (Bruker, Germany) from *P. aeruginosa* PAO1 Δ*murI* strain and its derivatives expressing deacylases, following the manufacturer’s instructions. Bacteria were grown on LB agar supplemented with 8 mM D-glutamate or LGA agar with 1 mM IPTG at 37°C for 24 hours. Then, 1-µL inoculation loop of bacteria was hydrolyzed for lipid extraction as previously described (Hernandez-Garcia et al., 2024). Mass spectra of lipid A were acquired using matrix-assisted laser desorption/ionization time-of-flight mass spectrometry (MALDI-TOF) in a MALDI Biotyper^®^ Sirius System (Bruker Daltonics, Inc.). Spectra were recorded in negative linear mode over a mass range of 500-3,000 *m/z*. The structural analysis was then performed using Clover MSDS software (Clover Bioanalytical Software, Granada, Spain).

### 2.6 RNA extraction and gene expression analysis

Total RNA was extracted from logarithmic-phase cultures using the High Pure RNA Isolation Kit (Roche Diagnostics, Germany) and adjusted to a concentration of 50 ng/μL. The relative expression levels of genes involved in lipid A modification (*eptA*, *lpxR*, *pagL* and *pagP*) were evaluated using two-step reverse transcription quantitative PCR (RT-qPCR). In the first step, reverse transcription of 200 ng of RNA was performed using random hexamer primers (60 μM) and the Transcriptor First Strand cDNA Synthesis kit (Roche Diagnostics, Germany). In the second step, 2 µL of the resulting cDNA was used in a 20 µL quantitative PCR reaction carried out on the LightCycler^®^ 480 Instrument II. Amplification was conducted using the LightCycler^®^ 480 Probes Master Kit (Roche Diagnostics, Germany), according to the supplier’s instructions. Gene-specific primers and optimal TaqMan probes from the Universal Probe Library (Roche, Germany) were used (**Table 2**). These primers were designed using the software provided by the University of the Sunshine Coast (Australia), accessible at https://primers.neoformit.com/. The relative gene expression was calculated using the Livak method (Livak and Schmittgen, 2001) and was normalized to the endogenous control gene *rpoS* of *P. aeruginosa* and the *aac(3)-IV* apramycin resistance gene encoded on the pMMB67EH-derived plasmids. All RNA extractions and RT-qPCR assays were performed in duplicate across four independent experiments.

### 2.7 TLR4 stimulation assays

HEK293-Blue TLR4 cells co-expressing either human (hTLR4) or murine (mTLR4) TLR4 complex and an NF-κB-inducible Secreted Embryonic Alkaline Phosphatase (SEAP) reporter (Invivogen) were cultured as recommended by the supplier. For TLR4 activation, 2.5 × 10^4^ cells per well were incubated with serial dilutions of either heat-killed bacterial cells for 17 hours at 37°C, 5% CO_2_ in a 96-well plate. Then, 20 μL of the supernatant from the stimulated cells was collected and incubated with 180 μL of the QUANTI-Blue™ reagent for 3 hours, and absorbance was measured at 630 nm, according to the manufacturer’s instructions (InvivoGen).

### 2.8 Animal experiments

Animal experiments were conducted in compliance with European Union guidelines (Directive 2010/63/EU) and current national legislation (RD 53/2013) on the protection of animals used for scientific purposes. All procedures received prior approval from the Animal Experimentation Ethics Committee of CHUAC (Project Reference Number 15002/2018/007). BALB/c mice (females, 7–10 weeks old; males for subcutaneous (SC) immunization), were housed in pathogen-free facilities at the Training Technology Center of the *Xerencia de Xestión Integrada A Coruña* (CTF-XXIAC), and were provided with pelleted rodent chow and water *ad libitum*.

For mouse inoculations, bacterial cultures were incubated in the appropriate medium at 37°C with shaking for 16 hours. After this period, cultures were diluted 1:50 in fresh medium and allowed to grow to an OD_600_ of 0.7. Bacteria were harvested by centrifugation (5,000 × g, 15 min, 4°C), washed three times with sterile saline solution (0.9% NaCl), and then resuspended in saline solution to the required volume to achieve the concentration needed for *in vivo* administration. An aliquot of this suspension was plated on the appropriate culture medium and incubated overnight at 37°C. The following day, the number of viable bacteria administered per mouse was determined (**Supplementary Table S1**). For immunogenicity assessments of various vaccine candidates in a murine model, different administration routes were used: IN, intradermal (ID), intramuscular (IM) and SC. In all cases, mice were anesthetized with sevoflurane before each inoculation. Immunizations schedules were optimized according to the route of administration, as previously reported (Cabral et al., 2020): for the IN route, two doses were administered with a 14-day interval; for the ID and SC routes, a three-dose was applied with weekly intervals; in the combined IN and IM regimen, two IN doses were given 14 days apart, along with three weekly IM doses; with both routes aligned during the first and third weeks. Blood (around of 50 μL) was collected by puncturing the submandibular vein and allowed to clot for 15 min. The samples were then centrifuged (2,800 × g, 15 min, 4°C) to remove cellular components, and the resulting serum was stored at –80°C until use. Vaginal fluid lavage (VFL) samples were obtained by flushing with 50 μL of sterile saline. These samples were centrifuged (800 × g, 15 min, 4°C), and the supernatant was transferred to a new tube containing 5 μL of a 10× protease inhibitor cocktail (Sigma-Aldrich), and then stored at –80°C until use.

### 2.9 Acute lung infection model

To induce acute lung infection in BALB/c mice, animals were anesthetized via inhalation with sevoflurane and administered the hypervirulent *P. aeruginosa* PA14 strain (1 × 10^6^ CFU) via IN. To minimize expulsion of the infectious material and to promote the development of acute pneumonia, mice remained under anesthesia for an additional 2 min. The monitoring protocol involved daily observation and individual tracking of each mouse after each procedure. Parameters included body weight loss and clinical symptoms, such as inactivity or lack of response to stimuli, hunching, piloerection, tip-toe walking, lack of grooming, stereotypies, slow or labored breathing with nasal discharge, among others. Each indicator was assigned a score as follows: 0 indicated normality; 1, a slight deviation from normal; 2, a moderate deviation; and 3, a significant deviation. This scoring system allowed for the quantification of procedure severity. A humane endpoint was applied for mice with a severity score of 14 or higher, or experiencing irreversible body weight loss exceeding 25%, in accordance with previous guidelines (Hawkins et al., 2011; Morton and Griffiths, 1985). Euthanasia was conducted by administering a barbiturate overdose via IP injection of 100 μL thiopental (50 mg/mL, Braun).

### 2.10 ELISA

The levels of serum IgG and IgA in VLF were quantitatively determined with a whole-bacterial cell ELISA in accordance with the previously described protocol (Cabral et al., 2020; Fuentes-Valverde et al., 2022). Each well was coated with formalin-inactivated bacteria (1 × 10^7^ CFU) in 100 µL of 100 mM carbonate-bicarbonate buffer, pH 9.6, and incubated overnight at 4°C. Formalin-killed cells were prepared beforehand by incubation with 1% (v/v) paraformaldehyde a 37 °C under agitation for 2 hours and washing with sterile PBS. The horseradish peroxidase (HRP)-conjugated anti-mouse IgG (Sigma-Aldrich) or IgA (Bethyl Laboratories) diluted 1:5,000 in DMEM supplemented with 10% fetal bovine serum were used as the secondary antibodies.

### 2.11 Statistical analysis

Statistical analyses were performed using GraphPad Prism software package (version 6.01). Means were compared using Student’s *t* test with Welch’s correction and the multiple *t*-test applied the Holm-Sidak method was used for the comparison of body weight changes. For curve comparison, data from TLR4 stimulation assays were analyzed for statistical significance using two-way ANOVA (Dunnett’s correction for multiple comparison). The Kaplan-Meier survival analysis was assessed with the Mantel-Cox log-rank test. For comparisons between two groups, the nonparametric and unpaired Mann-Whitney *U* test was applied, and Kruskal-Wallis test for comparisons of three or more groups. *P* values of < 0.05 were considered statistically significant.

## 3 Results

### 3.1 Production of heterologous LPS-modifying enzymes in *P. aeruginosa* PAO1 Δ*murI*

LPS is a relevant immunostimulatory factor to the protective efficacy of attenuated vaccines, as it activates the host immune system. However, its strong biological activity can also lead to toxicity, resulting in reactogenic responses. Therefore, achieving an optimal balance between immune activation and tolerable reactogenicity is essential for the development of safe while effective whole-cell vaccines. We decided to engineer a novel vaccine strain that produce enriched lipid A forms with either fewer compared to the canonical toxic hexa-acylated lipid A, as these modifications result in poor activation of TLR4/MD-2 (Maeshima and Fernandez, 2013). To this end, we overexpressed PagL to ensure an enrichment of penta-acylated lipid A species by removing the 3OH-C10 acyl chain from position 3 (Ernst et al., 2006; Rutten et al., 2006) (**Figure 1B**). In addition, we produced exogenous LpxR from *S.* Typhimurium, which is expected to remove the acyloxy acyl residue from the 3’ position of lipid A (Reynolds et al., 2006) (**Figure 1C**). Both *lpxR*_ST_ and *pagL* genes were cloned into the broad host-range expression vector pMMB67EH under the IPTG-inducible promoter. The resulting plasmids were introduced into the PAO1 Δ*murI* strain, generating the derivatives PAO1 Δ*murI* p*lpxR*_ST_ and PAO1 Δ*murI* p*pagL*.

**Figure 2:**
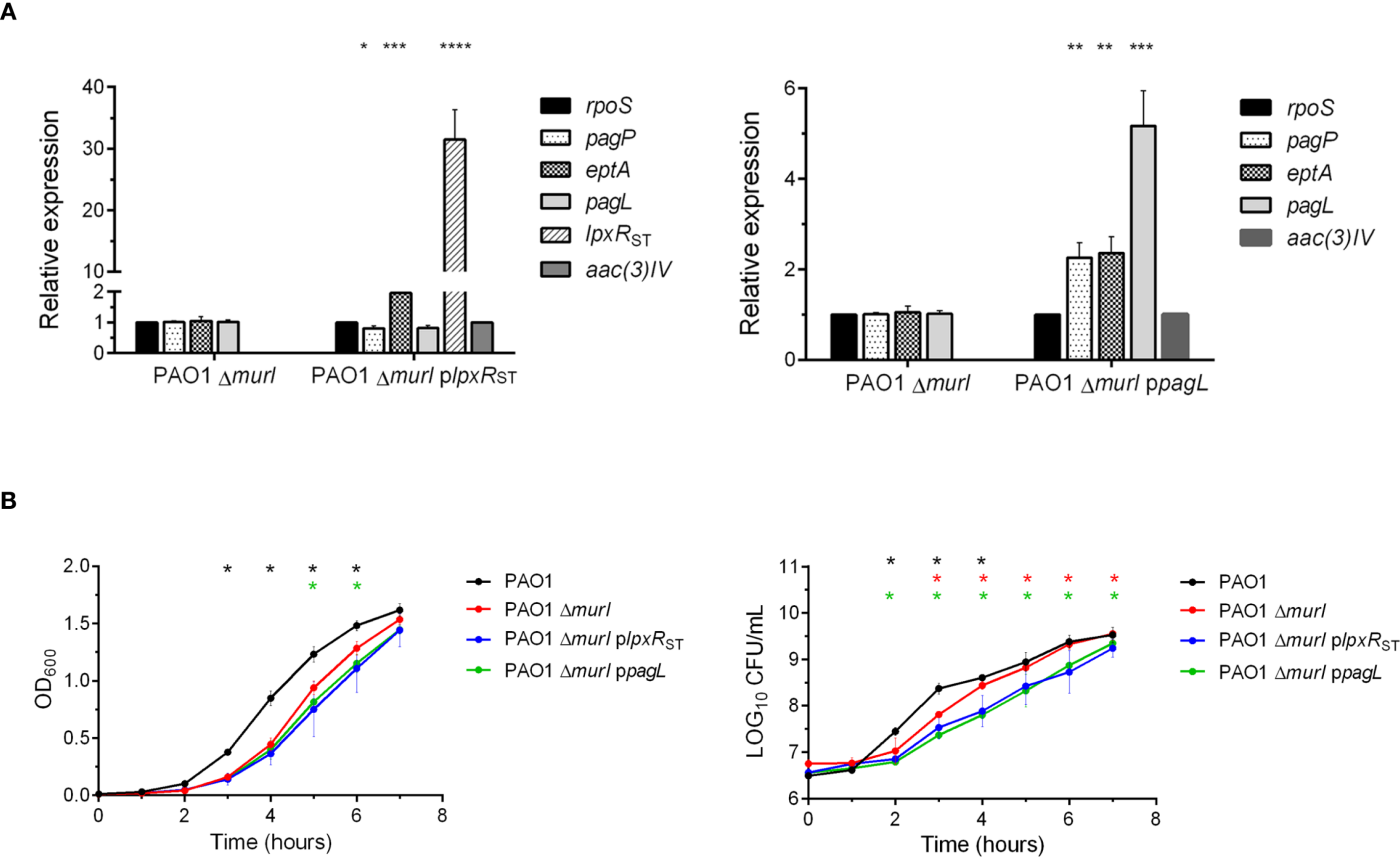
Gene expression and growth characteristics of LPS-modified *P. aeruginosa* strains. **(A)** Relative expression levels of genes involved in lipid A modification (*pagP*, *eptA*, *pagL* and *lpxR*) in the derivative strains PAO1 Δ*murI* p*lpxR*_ST_ and PAO1 Δ*murI* p*pagL* were analyzed by RT-qPCR and compared to the reference strain PAO1 Δ*murI*. Gene expression was quantified using the Livak method (2^−ΔΔCt^) and normalized to the housekeeping gene *rpoS* of *P. aeruginosa* and the *aac(3)-IV* gene present in the pMMB67EH-derived plasmids. Statistical significance was determined by unpaired *t*-test with Welch’s correction: *p < 0.05, **p < 0.01, ***p < 0.001, ****p < 0.0001. **(B)**. Growth and viability of *P. aeruginosa* PAO1, PAO1 Δ*murI* and its LPS-modified derivatives PAO1 Δ*murI* p*lpxR*_ST_ and PAO1 Δ*murI* p*pagL*. Cells were grown in LB medium supplemented with D-glutamate plus apramycin and IPTG, when required. Left panel: Growth was monitored by measuring culture turbidity (OD at 600 nm) at different times. Right panel: Culture viability (Log_10_ CFU per mL). Data represent the mean ± SD. *p < 0.01, unpaired *t*-test, compared to the parent strain PAO1 Δ*murI*.

The expression levels of *lpxR*_ST_ and *pagL* in both mutant derivatives and the parent strain were tested by RT-qPCR (**Figure 2A**). *rpoS* was used as housekeeping gene, and *aac(3)-IV* (apramycin resistance cassette) as a plasmid expression control. In the PAO1 Δ*murI* p*lpxR*_ST_ strain, *lpxR* expression was enhanced about 30-fold compared to PAO1 Δ*murI* and PAO1 Δ*murI* p*pagL*. Similarly, *pagL* expression was upregulated by about 5-fold in the PAO1 Δ*murI* p*pagL* strain relative to the other strains. We also examined the expression of other lipid A modifying enzymes in *P. aeruginosa*, such as *eptA* and *pagP*. Notably, a significant upregulation of *eptA* was observed in both the PAO1 Δ*murI* p*lpxR*_ST_ and PAO1 Δ*murI* p*pagL* strains. Furthermore, *pagP* was upregulated about two-fold in PAO1 Δ*murI* p*pagL* strain. In contrast, a slight reduction in *pagP* and *pagL* expression was detected in the PAO1 Δ*murI* p*lpxR*_ST_ strain. Together, these results confirm the successful expression of the targeted genes, and show that their expression influences that of other LPS-modifying enzymes. Next, we evaluate whether the activity of these enzymes on lipid A affected the growth and viability of the bacterial strains. To do this, we compared their growth kinetics in LGA medium supplemented with IPTG. Compared to the PAO1 Δ*murI*, both mutant derivatives exhibited a slight delay in the exponential growth phase relative to the parent strain (**Figure 2B**, left panel), which was associated with a modest but significant decrease in the number of viable bacteria (**Figure 2B**, right panel).

### 3.2 Analysis of recombinant lipid A structures

**Figure 3:**
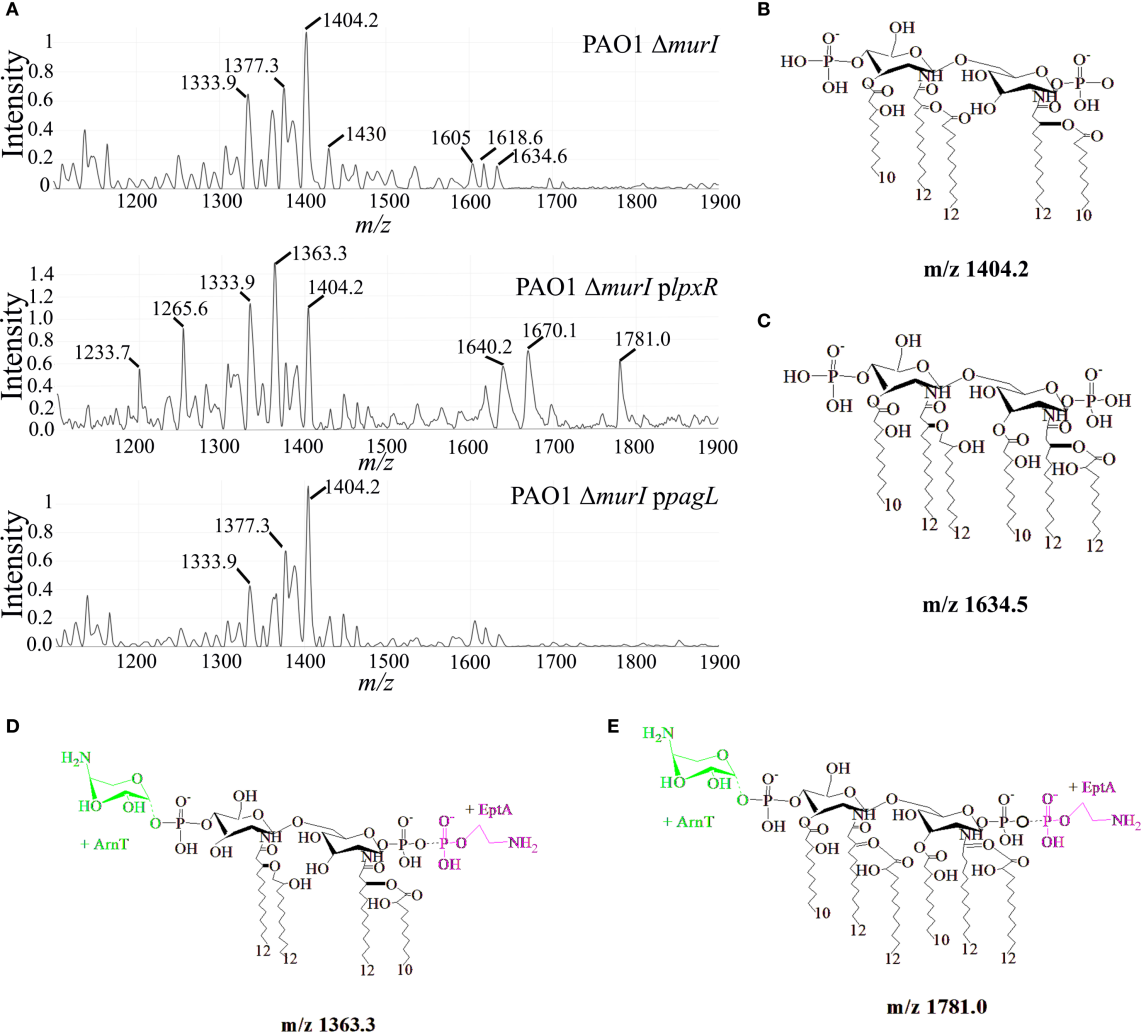
Structural analysis of lipid A in *P. aeruginosa* PAO1 Δ*murI*, PAO1 Δ*murI* p*lpxR*_ST_, and PAO1 Δ*murI* p*pagL*. **(A)** Purified lipid A was analyzed by matrix-assisted laser desorption/ionization time-of-flight mass spectrometry (MALDI-TOF), and negative-ion mode spectra are shown. **(B-E)** Proposed structures corresponding to selected *m/z* peaks annotated in the spectra shown in panel **(A)** Structures were generated using ChemDraw software.

Lipid A was extracted from exponentially growing cultures of *P. aeruginosa* PAO1 Δ*murI* and their mutant derivatives grown in the presence of IPTG and antibiotics, and analyzed using MALDI-TOF in negative linear mode. An ample range of peaks from *m/z* 1200 to 1800 was detected across all strains, but their number and relative abundance varied considerably (**Figure 3A**). In *P. aeruginosa* PAO1 Δ*murI*, the most predominant lipid A species appeared at *m/z* 1404.2. This peak may correspond to a penta-acylated, bis-phosphorylated lipid A with one 3OH-C10 and two 3OH-C12 primary acyl chains, one C10:O and one C12:O secondary acyl chains (**Figure 3B**) (Hernández-García et al., 2024). This result partially differs from that reported by Ernst et al. (2006), who identified the major lipid A species of PAO1 lipid A at *m/z* 1447 (Ernst et al., 2006). This discrepancy may be explained by the substitution of a C12 secondary acyl chain at position 2 with a C10 acyl chain in our vaccine strain. Incorporation of the C12 secondary acyl chain at this position is catalyzed by LpxL2 (also known as HtrB2), which, however, showed high specificity for C12 substrates in PAO1 (Hittle et al., 2015). Recent studies have reported lipid A species at *m/z* 1403 in clinical *P. aeruginosa* isolates harboring mutations in *lpxL2*, *acrB2* or *cbrA* (Hernández-García et al., 2024), suggesting that our vaccine strain may harbor additional mutations beyond *murI*. In addition, the presence of this prominent species reflects the strong activity of PagL, and the lack of activity of dioxygenase enzymes: LpxO1 and LpxO2. Additionally, a broad set of low-abundance peaks between *m/z* 1430.9 and 1534.3 were observed. These likely correspond to penta-acylated lipid A species with two C12:O secondary acyl chains, possibly differencing in their hydroxylation status (*e.g. m/z* 1430.9, 1447.6, 1463.4, respectively). Of note that peaks corresponding with lipid A species harboring also C12 secondary acyl chains at 2 position were also detected in clinical isolates harboring mutations in *lpxL2*, *acrB2* or *cbrA* (Hernández-García et al., 2024), suggesting a reduced of specificity of LpxL2. Some of these peaks may carry additional modifications, such as Ara4N or pEtN, presumably at the 1 and/or 4´phosphate groups, resulting in peaks at *m/z* 1564, 1578, respectively. In addition, four abundant peaks with slightly lower *m/z* values (ranging from 1333.9 to 1377.3) were detected, presumably corresponding to dephosphorylated variants of the major penta-acylated structure (*e.g.*, *m/z* 1377.3), either tetra-acylated species lacking both secondary acyl chains and AraN4 groups (*e.g.*, *m/z* 1333.9). Furthermore, a minor group of peaks at *m/z* 1605, 1618.6 and 1634.6 were detected, and likely corresponds to hexa-acylated lipid A species. For example, the *m/z* 1634.6 peak may represent a bis-phosphorylated hexa-acylated species with four hydroxylated acyl chains, while the reduced mass observed for the peaks at 1618 and 1605 suggest loss of hydroxyl groups (**Figure 3C**).

The expression of *lpxR_ST_* in *P. aeruginosa* PAO1 Δ*murI* resulted in a substantial increase in the abundance of tetra-acylated lipid A species (*m/z* 1233.7-1363.3). The most prominent among these was the peak at *m/z* 1363.3, which likely corresponds to a tetra-acylated lipid A form with two primaries 3OH-C12 acyl-chains at 2 and 2´positions, two secondary acyl chains and an Ara4N and pEtN groups (**Figure 3D**). The synthesis of this species may reflect the combined activity of LpxR, PagL and EptA (**Figure 2A**). The remaining peaks may reflect structural variants of this tetra-acylated form, resulting from the loss or substitution of polar groups at positions 1 and 4´ (*e.g.*, *m/z* 1265.6). Curiously, two additional peaks of moderate abundance appeared at *m/z* 1670.1 and 1781.0, presumably corresponding with novel hexa-acylated forms with pEtN and lacking one phosphate group (*m/z* 1670.1), or additionally modified with Ara4N (*m/z* 1781) (**Figure 3E**). The emergence of these species may reflect the absence of PagL activity and the upregulation of pEtN modification pathways, in agreement with results from gene expression analysis (**Figure 2A**). The expression of *pagL* in *P. aeruginosa* PAO1 Δ*murI* led to an increased relative abundance of the peak at *m/z* 1404.2, and a subsequent reduction of hexa-acylated forms, which may in part be attributed to enhanced activity of PagL. Together, MS spectrometry analysis shows that LpxR and PagL expression in *P. aeruginosa* PAO1 Δ*murI* varies the heterogeneity and abundance of lipid A species which may have implications in their biological activity.

### 3.3 Stimulation assays of TLR4

**Figure 4:**
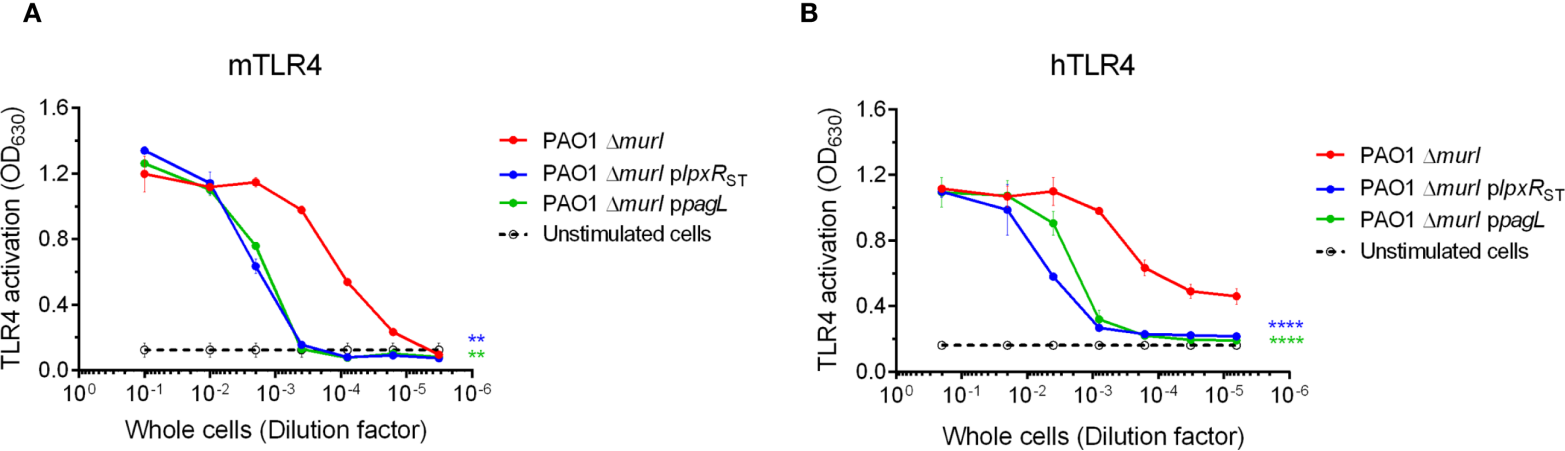
TLR4 activation by whole-cell preparations of PAO1 Δ*murI* and its derivatives with enzymatically modified lipid A by LpxR_ST_ or PagL. HEK293-Blue cells expressing mTLR4 **(A)** or hTLR4 **(B)** were cultured in microtiter plates and stimulated with 1:10 serial dilutions of heat-inactivated whole-cells (initial OD_600_ = 0.1) for 17 hours at 37°C, 5% CO_2_. Alkaline phosphatase activity in the culture supernatants was measured at OD_630_. Results represent the mean ± SD of three independent experiments. **p < 0.01, ****p < 0.0001 (two-way ANOVA with Dunnett’s multiple comparisons test), relative to the parent strain.

We then investigated whether the altered LPS profiles in the mutant derivatives affected TLR4 signaling. To this end, NF-κB SEAP reporter HEK293 cells expressing the murine TLR4/MD-2/CD14 complex were stimulated with heat-inactivated whole-cell bacteria preparations of PAO1 Δ*murI* and its derivatives. TLR4 activation was evaluated by measuring SEAP reporter gene expression. Interestingly, both mutant strains stimulated approximately 100-fold lower mTLR4 activation compared to the parental strain (**Figure 4A**). This reduction is likely due to increased proportion of tetra-acylated lipid A species, known as TLR4 antagonist, in PAO1 Δ*murI* p*lpxR*_ST_, and a decrease in highly immunostimulatory hexa-acylated species in PAO1 Δ*murI* p*pagL*, resulting in an overall reduction in whole-cell bacterial activity. Because human and murine TLR4 respond differently to various LPS structures and given that our vaccine is intended for human use, we next assessed hTLR4 activation. PAO1 Δ*murI* p*lpxR*_ST_ stimulated hTLR4 approximately 100-fold less than the parental strain, while PAO1 Δ*murI* p*pagL* showed a milder reduction between 10 and 100-fold depending on the dose (**Figure 4B**). Together, these data suggest that both recombinant strains exhibit reduced TLR4-mediated activity, indicating a potential decrease in LPS-associated toxicity compared to the wild-type vaccine strain.

### 3.4 Assessment of vaccine efficacy in experimental animals

In spite LPS cause reactogenic reactions in attenuated vaccines, it plays a relevant role in the immunogenic response. Therefore, reducing LPS activity could potentially compromise the efficacy of our vaccine strain. Moreover, a reduction in the TLR4 activation may not suffices to reduce the vaccine toxicity *in vivo*. To address both concerns, we evaluated the protective capacity and toxic activity of the novel recombinant strains in a murine model.

**Figure 5:**
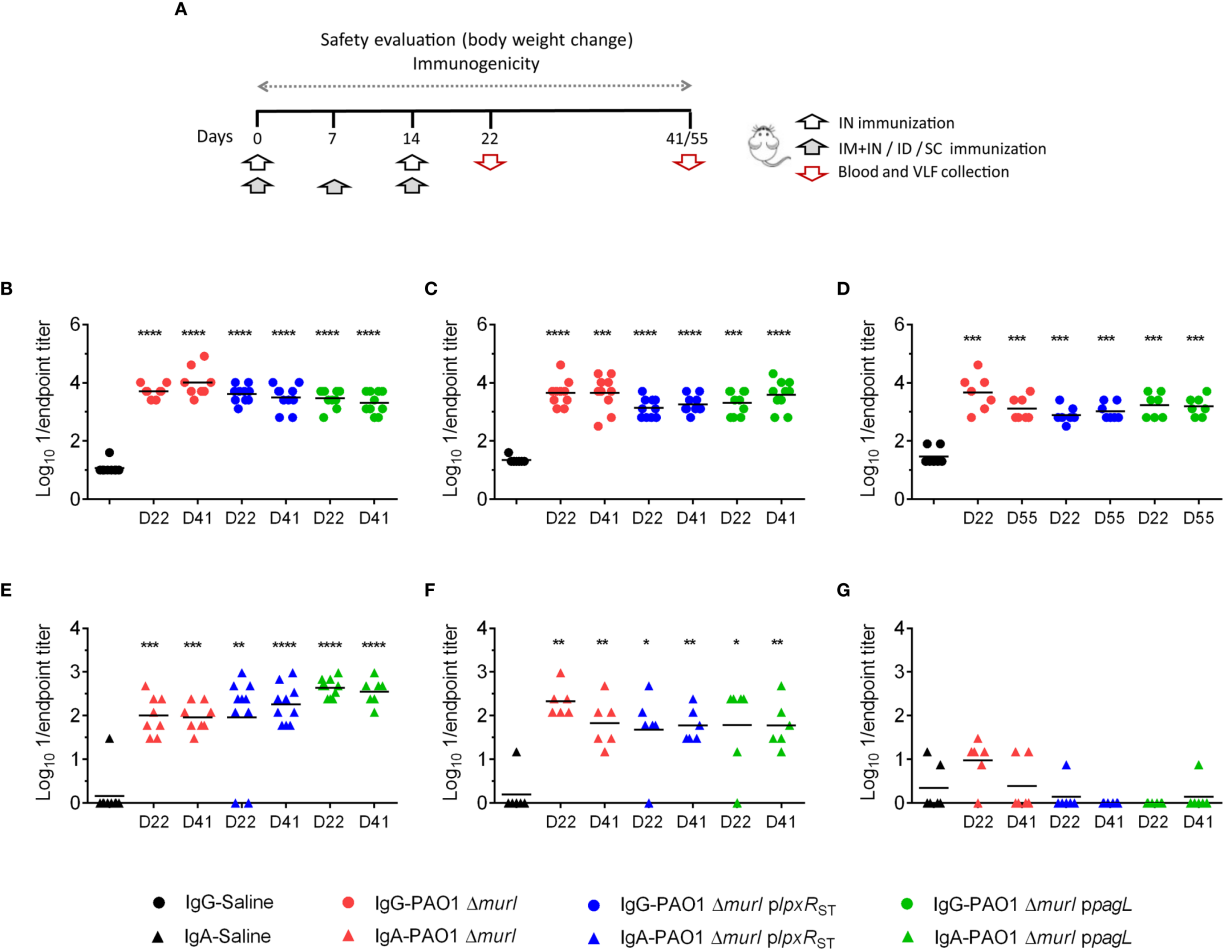
Systemic and mucosal antibody-mediated immune response against PAO1 in BALB/c mice after immunization with PAO1 Δ*murI* and its lipid A variants. **(A)** Immunization schedule based on the administration routes used. Determination of serum IgG **(B-D)** and vaginal wash IgA **(E-G)** titers against PAO1 by ELISA at 7 days (D22) and 4 to 6 weeks (D41-D55) after the final vaccine dose. Serum IgG titers (circles) following IN **(B)**, ID **(C)**, and SC **(D)** immunizations. Vaginal fluid lavage IgA titers (triangles) following IN **(E)**, IN+IM **(F)**, and ID **(G)** immunizations. *p < 0.05, **p < 0.01, ***p < 0.001, ****p < 0.0001 (Mann-Whitney test), compared to the saline group.

To evaluate the immunogenic capacity of PAO1 Δ*murI* p*lpxR*_ST_ and PAO1 Δ*murI* p*pagL* relative to the parent strain, mice were immunized with each of the three strains, with PBS as a control, via different routes: IN, a combination of IN and IM, ID, and SC (**Supplementary Table S1**). The combined IN and IM routes was designed to balance mucosal immunity with overall protection by using lower, and therefore potentially safer, doses via the IN route (Cabral et al., 2020). Blood and VFL samples were collected 7 days after the final vaccine dose (day 22, D22), and again between 4 to 6 weeks post-vaccination (D41-D55) (**Figure 5A**), and antibody titers were determined by whole-cell ELISA. Interestingly, all vaccine candidates elicited robust serum IgG responses across all administration routes tested (IN, ID and SC) (**Figures 5B–D**). However, elevated IgA levels were detected only in VFL samples from mice vaccinated via the IN route and the combined IN plus IM route (**Figures 5E, F**). Unexpectedly, IN administration of PAO1 Δ*murI* p*pagL* resulted in significantly higher IgA titers compared to PAO1 Δ*murI*, both at D22 (*p* = 0.0036) and D41 (*p* = 0.0059) of the vaccination schedule (**Figure 5E**). No significant differences were detected in ID immunizations (**Figure 5G**).

**Figure 6:**
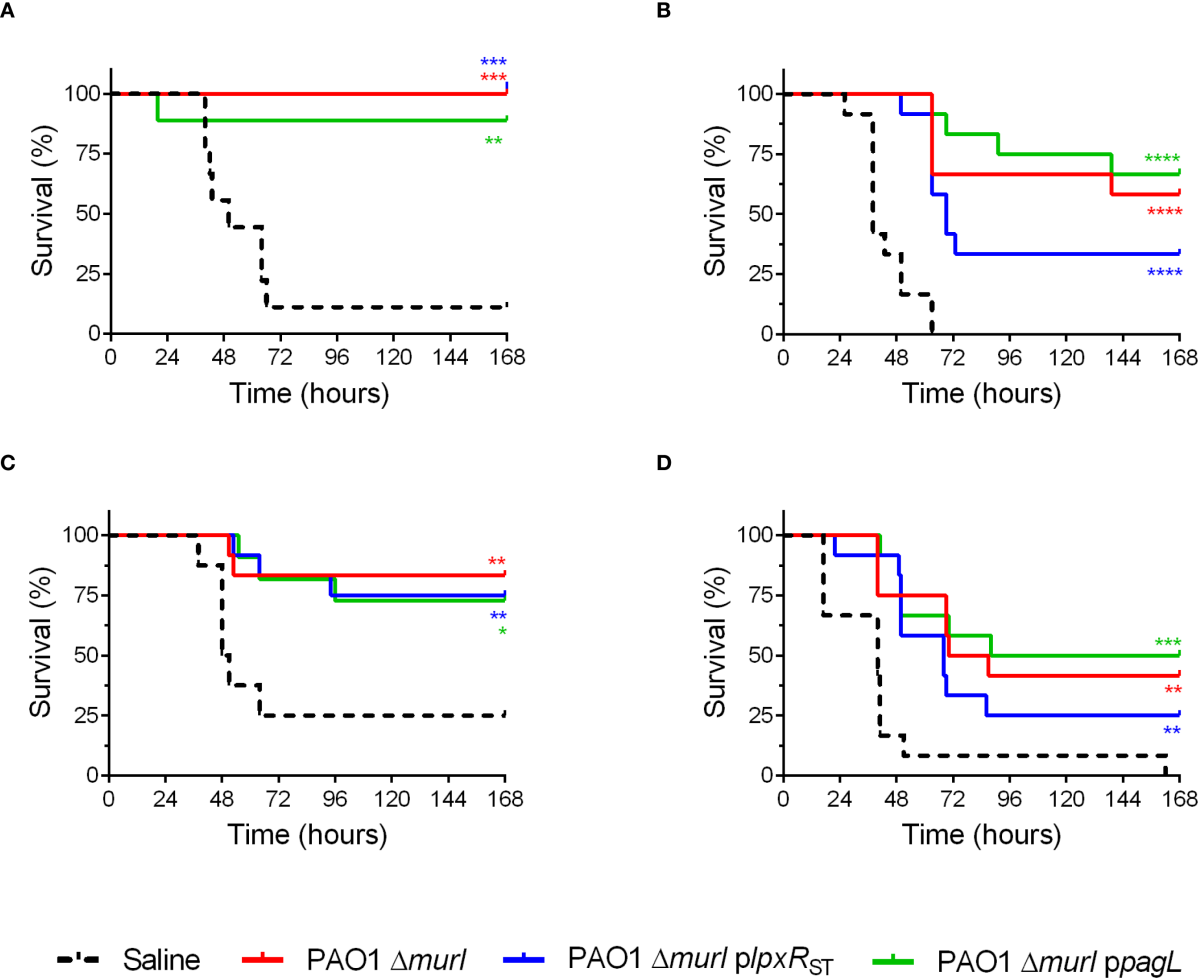
Protection against acute pneumonia after immunization with PAO1 Δ*murI* and its lipid A derivatives expressing the deacylases LpxR_ST_ or PagL. BALB/c mice (*n* = 9-12) were immunized via different delivery routes: IN **(A)**, IN+IM **(B)**, ID **(C)**, and SC **(D)**. Mice were IN challenged with PA14 (1 × 10^6^ CFU) 4–6 weeks after last immunization. Survival rates of vaccinated versus sham-immunized mice are shown. *p < 0.05, **p < 0.01, ***p < 0.001, ****p < 0.0001 (Log-rank Mantel-Cox test), compared to the saline control group.

To assess vaccine efficacy, animals were immunized via IN, IM or a combination of routes as described in **Figure 5A**. Then, mice were IN challenged with the virulent *P. aeruginosa* PAO1 strain. Survival rates of the vaccinated mice were compared to those of an unvaccinated control group. Protection varied according to the route of vaccine administration. Mice immunized IN showed the highest survival rate (87.5%), whereas those vaccinated subcutaneously exhibited lower survival, ranging from 30% to 50% (**Figures 6A–D**). No statistically significant differences in survival were observed among the groups vaccinated with the different recombinant strains. However, mice vaccinated with PAO1 Δ*murI* p*pagL* via IN plus IM or SC routes showed relatively higher survival compared to those vaccinated with PAO1 Δ*murI* p*lpxR*_ST_ (**Figures 6B, D**). For example, at 72 hours post challenge, mice immunized with PAO1 Δ*murI* p*pagL* showed survival rates of 58-83%, whereas those immunized with PAO1 Δ*murI* p*lpxR*_ST_ showed survival rates of 33-42%. Together, our data reflects that vaccine efficacy was not compromised by the overproduction of LpxR_ST_ or PagL.

### 3.5 Safety assessment of vaccine candidates in experimental animals

**Figure 7:**
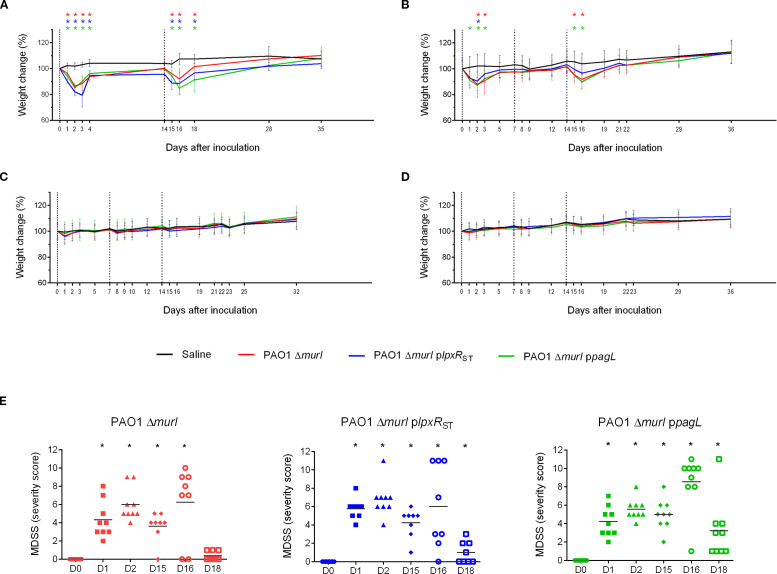
Vaccine safety assessment in BALB/c mice following administration of the PAO1 Δ*murI* strain or its lipid A variants expressing LpxR_ST_ or PagL. Effect of immunization on body weight following two IN doses **(A)**, a combined IN and IM regimen **(B)**, or three doses by ID **(C)** or SC **(D)** routes. The graph shows the percentage change in the average body weight of mice across experimental groups. Dotted lines indicate immunization days: day 0 (start of the experiment), day 7, and day 14. Data represent the mean ± SD. *p < 0.05, unpaired *t*-test, compared to the saline group. **(E)** Clinical evaluation following IN administration of the PAO1 Δ*murI* strain and its LPS-modified derivatives expressing LpxR_ST_ or PagL deacylases. Clinical assessments in BALB/c mice were performed using a scoring system considering parameters such as piloerection, grooming, mobility, respiratory pattern, dehydration, and body weight. D, day. *p < 0.05, unpaired *t*-test.

The reactogenicity of the recombinant strains was evaluated in immunized animals (**Figure 5A**) by monitoring body weight and clinical signs after immunization. Mice vaccinated via the IN route generally exhibited a significant decrease in body weight in some cases approaching a 20% reduction after the first vaccine dose, mainly PAO1 Δ*murI* p*lpxR*_ST_ strain (**Figure 7A**). These weight losses were transient, and most mice regained their initial weight within 7 days post-inoculation. In contrast, in the combined IN and IM immunization regimen, weight loss occurred only after IN administration (**Figure 7B**), while no adverse effects were detected after IM administration alone. Mice inoculated via ID and SC (**Figures 7C, D**) routes showed only minor weight fluctuations, with decreases under 5% relative to the initial group weight.

Mice vaccinated via the IN route generally displayed mild clinical signs, including a slightly unkempt appearance, piloerection, and in some cases, a hunched posture with reduced mobility. However, respiratory function remained unaffected, and no episodes of dyspnea were observed. No adverse effects were detected after IM administration alone, neither significant behavioral changes were observed. However, small skin lesions were observed at injection sites in mice receiving ID inoculations. Severity scores calculated across strains and time points revealed no notable differences between the recombinant strains and the wild type (**Figure 7E**).

## 4 Discussion

*P. aeruginosa* is a major respiratory pathogen, prompting efforts to develop effective antimicrobials and vaccines (Killough et al., 2022). Live attenuated vaccines, particularly auxotrophic strains, are promising due to their high immunogenicity and ability to elicit robust cellular responses. Deletion of the *murI* gene in *P. aeruginosa* PAO1 resulted in a D-glutamate auxotroph that exhibits strictly self-limiting growth *in vivo* and undergoes autolysis in its absence, thereby preventing infection while inducing protective and cross-reactive immune responses (Cabral et al., 2017). This intrinsic replication-limiting property clearly distinguishes our candidate from other attenuated strains that retain broader replicative capacity *in vivo*, and it provides a strong additional safety margin. However, high-dose IN administration caused significant weight loss in mice (Cabral et al., 2020; Fuentes-Valverde et al., 2022), likely due to the endotoxin-like activity of LPS. To reduce this reactogenicity, we engineered vaccine strains with modified lipid A by overexpressing the deacylases LpxR from *S.* Typhimurium and PagL from *P. aeruginosa*, aiming to reduce TLR4 activation. We anticipate comparable safety and efficacy profiles for the auxotrophic derivatives engineered to overexpress *pagL* and *lpxR*. With regard to the potential increase in β-lactam and polymyxin B resistances associated with *pagL* overexpression, we believe the auxotrophic nature of our vaccine strain, together with the demonstrated lack of systemic persistence, substantially mitigates the associated risks.

LPS plays a crucial physiological role in stabilizing the outer membrane by forming ionic bridges with Ca²^+^ and Mg²^+^ cations (Knirel et al., 2006). During host-pathogen interaction, lipid A is delivered to the TLR4/MD-2 complex by CD14 and the LPS-binding protein. Within this complex, the acyl chains of lipid A establish hydrophobic interactions with aliphatic amino acids, while its phosphate and hydroxyl groups form hydrogen bonding with lysines (Park et al., 2009). The immunostimulatory potency of LPS depends on lipid A acylation, for example, the hexa-acylated lipid A form is approximately 100-fold more active than the penta-acylated form (Park et al., 2009). Our spectral analysis of vaccine strains with native LPS revealed considerable heterogeneity, including both penta- and hexa-acylated species.

Production of LpxR in PAO1 Δ*murI* produced an enrichment of tetra-acylated and hexa-acylated species decorated with Ara4N and pEtN groups. In *E. coli*, LpxR expression generated, among other species, a tetra-acylated form (R-lipid A) that was approximately 30-fold less active in stimulating hTLR4 than native hexa-acylated (U-lipid A), but 30- to 100-fold more active than the hepta-acylated variant (P-lipid A) produced through *pagP* expression (Kawasaki et al., 2012). Hence, the authors suggested that LpxR-mediated 3’-*O*-deacylation and PagP-dependent palmitoylation act synergistically in *S.* Typhimurium to modulate lipid A bioactivity. Thus, the alterations detected in our mutant may cause by a reduced PagL and PagP activity, together with upregulation of pEtN modification pathways in agreement with that detected in RT-qPCR assays. In contrast to *Enterobacteriaceae*, where PagP introduces a palmitate at the 2’ position, *P. aeruginosa* PagP (PA1343) adds a secondary acyl chain at the 3’ position of lipid A (Thaipisuttikul et al., 2014). This indicates potential competition between LpxR_ST_ and PagP for the same modification site on the LPS molecule in *P. aeruginosa* (**Figure 1**). Moreover, heterologous expression of *lpxR*_ST_ in *P. aeruginosa* alters lipid A composition by increasing *eptA* expression, while slightly reducing *pagP* and *pagL* levels. This suggests a regulatory adjustment of these enzymes at reinforcing membrane charge and maintaining a balance of lipid A remodeling. Notably, stimulation with heat-killed PAO1 Δ*murI* p*lpxR*_ST_ cells resulted in a 100-fold decrease in hTLR4 activation compared to native PAO1 (**Figure 4B**), highlighting its potential as a safer vaccine candidate.

In addition, we also tested if enhancement of PagL expression may reduce the number of active hexa-acylated species present in the wild type leading to reduced LPS activity (**Figure 1B**). Overexpression of *pagL* in *Bordetella pertussis* and *E. coli* reduces hTLR4 activation by generating modified LPS (Geurtsen et al., 2006; Kawasaki et al., 2004). In our *P. aeruginosa* vaccine strain, production of PagL revealed a low frequency of penta- and hexa-acylated variants as compared to the wild type (**Figure 3**), indicating altered acylation patterns. This change likely triggers membrane stress responses via the PhoPQ or PmrAB two component systems (Simpson and Trent, 2019), leading to *eptA* and *pagP* upregulation to restore membrane integrity. These modifications may interfere with TLR4/MD-2 recognition, particularly at the phosphate moieties, reducing TLR4 activation. Stimulation with heat-inactivated PAO1 Δ*murI* p*pagL* cells resulted in significantly lower mTLR4 and hTLR4 activation than wild-type PAO1 strain, suggesting again reduced reactogenicity of our vaccines. Interestingly, in *B. pertussis*, *pagL* overexpression reduce IL-6 levels in response to purified LPS, but increases activation with whole cells. This effect may result from a higher availability of free lipid A, as membrane-associated lipid A establishes fewer Van der Waals interactions, weakening its binding and promoting membrane evagination and enhanced outer membrane vesicles (OMV) production (Geurtsen et al., 2005). Similarly, studies have shown that PagL-mediated deacylation increases both the number and size of OMVs (Elhenawy et al., 2016). Furthermore, PhoPQ and PmrAB activation, under acidic pH or low Mg^2+^ concentration promotes OMV release enriched with hepta-acylated lipid A via PagP (Bonnington and Kuehn, 2016).

Identifying correlations between vaccine induced protection and biological indicators (biomarkers) is relevant for predicting efficacy and comparing formulations. In acute *P. aeruginosa* infection models, the humoral immune response plays a critical role in reducing pulmonary bacterial loads following IN administration of *P. aeruginosa* (Sen-Kilic et al., 2021). Previous work showed that immunization with PAO1 Δ*murI* induced serum IgG capable of recognizing heterologous *P. aeruginosa* strains, and passive antibody transfer conferred protection in mice (Cabral et al., 2017, 2020). In this study, although minor variations in administered CFU doses within the same order of magnitude, as well as differences in administration route and schedule, could potentially influence immune outcomes, our data show that all vaccine candidates (via IN, ID, SC) elicited comparable increases in serum IgG levels. Notably, IN immunization with PAO1 enhanced mucosal IgA levels in both bronchoalveolar and vaginal washes (Cabral et al., 2020). A similar increase in vaginal IgA was observed with our enzyme-overexpression strains, suggesting that coordinated mucosal responses across different mucosal sites (Belyakov and Ahlers, 2009) are preserved in these mutants. Previous studies have also reported elevated total and pathogen-specific IgA during early CF infection (Collin et al., 2020), along with increased PagL expression in *P. aeruginosa* isolates from infants with CF (Ernst et al., 2006). However, PagL function can be lost during long-term airway adaptation, correlating with more severe disease (Ernst et al., 2007). In agreement with these observations, the PagL-overexpressing strain in our study induced slightly higher mucosal IgA despite reduced TLR4 activation. We hypothesize that attenuated inflammation may minimize tissue damage, facilitating dendritic cell interactions and promoting a cytokine milieu conducive to IgA class switching. Alternatively, PagL-modified lipid A may bias TLR4 signaling toward TRIF, as reported for monophosphoryl lipid A (Mata-Haro et al., 2007), thereby dampening pro-inflammatory cytokines and favoring a regulatory environment (IL-10, TGF-β) that supports IgA induction (Bagheri et al., 2019). Indeed, all mutants induced robust antibody responses, including IgG and IgA levels, and protected mice against acute lung infection caused by *P. aeruginosa*. However, protection outcomes varied noticeably by route of administration: IN immunization yielded the highest survival rates, whereas SC vaccination was associated with lower survival. Yet, our mutants failed to reduce vaccine reactogenicity *in vivo*, despite diminished TLR4 activation observed *in vitro*. This discrepancy suggests that additional immune pathways beyond TLR4 may contribute significantly to the inflammatory response, including the activation of other pattern recognition receptors, less effective lipid A modifications generated under host physiological conditions, or the involvement of non-LPS components. Our findings underscore a critical, route-dependent trade-off between vaccine efficacy and tolerability. While IN administration provided the strongest protection against a lung challenge, this benefit was counterbalanced by significant reactogenicity at the mucosal site. Conversely, systemic routes such as SC or ID injection were well tolerated, but conferred substantially weaker protection. This dichotomy represents a major obstacle for clinical translation. Achieving an optimal balance between safety and efficacy will likely require strategies such as detoxified adjuvants, prime-boost regimens that separate systemic priming from mucosal boosting, or novel formulations that minimize epithelial irritation while preserving mucosal immune engagement. Complementary to these approaches, future research should explore additional lipid A-modifying enzymes, extending the modifications generated in this study, as a means to develop safer and less reactogenic anti-*P. aeruginosa* vaccines.

## Data Availability

The original contributions presented in the study are included in the article/**Supplementary Material.** Further inquiries can be directed to the corresponding authors.

## References

[B1] Arenas J. Pupo E. Phielix C. David D. Zariri A. Zamyatina A.. (2020). Shortening the lipid A acyl chains of *Bordetella pertussis* enables depletion of lipopolysaccharide endotoxic activity. Vaccines (Basel). 8, 594. doi: 10.3390/vaccines8040594, PMID: 33050234 PMC7712016

[B2] Bagheri Y. Babaha F. Falak R. Yazdani R. Azizi G. Sadri M.. (2019). IL-10 induces TGF-beta secretion, TGF-beta receptor II upregulation, and IgA secretion in B cells. Eur. Cytokine Netw. 30, 107–113. doi: 10.1684/ecn.2019.0434, PMID: 31957700

[B3] Belyakov I. M. Ahlers J. D. (2009). What role does the route of immunization play in the generation of protective immunity against mucosal pathogens? J. Immunol. 183, 6883–6892. doi: 10.4049/jimmunol.0901466, PMID: 19923474

[B4] Bonnington K. E. Kuehn M. J. (2016). Outer membrane vesicle production facilitates LPS remodeling and outer membrane maintenance in *Salmonella* during environmental transitions. mBio. 7, e01532-16. doi: 10.1128/mBio.01532-16, PMID: 27795394 PMC5082901

[B5] Cabral M. P. Correia A. Vilanova M. Gartner F. Moscoso M. García P.. (2020). A live auxotrophic vaccine confers mucosal immunity and protection against lethal pneumonia caused by *Pseudomonas aeruginosa* . PloS Pathog. 16, e1008311. doi: 10.1371/journal.ppat.1008311, PMID: 32040500 PMC7034913

[B6] Cabral M. P. García P. Beceiro A. Rumbo C. Pérez A. Moscoso M.. (2017). Design of live attenuated bacterial vaccines based on D-glutamate auxotrophy. Nat. Commun. 8, 15480. doi: 10.1038/ncomms15480, PMID: 28548079 PMC5458566

[B7] Cigana C. Curcuru L. Leone M. R. Ierano T. Lore N. I. Bianconi I.. (2009). *Pseudomonas aeruginosa* exploits lipid A and muropeptides modification as a strategy to lower innate immunity during cystic fibrosis lung infection. PloS One 4, e8439. doi: 10.1371/journal.pone.0008439, PMID: 20037649 PMC2793027

[B8] Collaborators, G. B. D. A. R (2022). Global mortality associated with 33 bacterial pathogens in 2019: a systematic analysis for the Global Burden of Disease Study 2019. Lancet 400, 2221–2248. doi: 10.1016/S0140-6736(22)02185-7, PMID: 36423648 PMC9763654

[B9] Collin A. M. Lecocq M. Noel S. Detry B. Carlier F. M. Aboubakar Nana F.. (2020). Lung immunoglobulin A immunity dysregulation in cystic fibrosis. EBioMedicine 60, 102974. doi: 10.1016/j.ebiom.2020.102974, PMID: 32927272 PMC7495088

[B10] De Oliveira D. M. P. Forde B. M. Kidd T. J. Harris P. N. A. Schembri M. A. Beatson S. A.. (2020). Antimicrobial resistance in ESKAPE pathogens. Clin. Microbiol. Rev. 33, e00181-19. doi: 10.1128/CMR.00181-19, PMID: 32404435 PMC7227449

[B11] Dower W. J. Miller J. F. Ragsdale C. W. (1988). High efficiency transformation of *E. coli* by high voltage electroporation. Nucleic Acids Res. 16, 6127–6145. doi: 10.1093/nar/16.13.6127, PMID: 3041370 PMC336852

[B12] Elhenawy W. Bording-Jorgensen M. Valguarnera E. Haurat M. F. Wine E. Feldman M. F. (2016). LPS remodeling triggers formation of outer membrane vesicles in *Salmonella* . mBio 7, e00940-16. doi: 10.1128/mBio.00940-16, PMID: 27406567 PMC4958258

[B13] Ernst R. K. Adams K. N. Moskowitz S. M. Kraig G. M. Kawasaki K. Stead C. M.. (2006). The *Pseudomonas aeruginosa* lipid A deacylase: selection for expression and loss within the cystic fibrosis airway. J. Bacteriol 188, 191–201. doi: 10.1128/JB.188.1.191-201.2006, PMID: 16352835 PMC1317579

[B14] Ernst R. K. Moskowitz S. M. Emerson J. C. Kraig G. M. Adams K. N. Harvey M. D.. (2007). Unique lipid a modifications in *Pseudomonas aeruginosa* isolated from the airways of patients with cystic fibrosis. J. Infect. Dis. 196, 1088–1092. doi: 10.1086/521367, PMID: 17763333 PMC2723782

[B15] Ernst R. K. Yi E. C. Guo L. Lim K. B. Burns J. L. Hackett M.. (1999). Specific lipopolysaccharide found in cystic fibrosis airway *Pseudomonas aeruginosa* . Science 286, 1561–1565. doi: 10.1126/science.286.5444.1561, PMID: 10567263

[B16] Fuentes-Valverde V. García P. Moscoso M. Bou G. (2022). Double auxotrophy to improve the safety of a live anti-*pseudomonas aeruginosa* vaccine. Vaccines (Basel). 10, 1622. doi: 10.3390/vaccines10101622, PMID: 36298487 PMC9610692

[B17] Geurtsen J. Steeghs L. Hamstra H. J. Ten Hove J. de Haan A. Kuipers B.. (2006). Expression of the lipopolysaccharide-modifying enzymes PagP and PagL modulates the endotoxic activity of *Bordetella pertussis* . Infect. Immun. 74, 5574–5585. doi: 10.1128/IAI.00834-06, PMID: 16988232 PMC1594925

[B18] Geurtsen J. Steeghs L. Hove J. T. van der Ley P. Tommassen J. (2005). Dissemination of lipid A deacylases (*pagL*) among gram-negative bacteria: identification of active-site histidine and serine residues. J. Biol. Chem. 280, 8248–8259. doi: 10.1074/jbc.M414235200, PMID: 15611102

[B19] Gust B. Challis G. L. Fowler K. Kieser T. Chater K. F. (2003). PCR-targeted *Streptomyces* gene replacement identifies a protein domain needed for biosynthesis of the sesquiterpene soil odor geosmin. Proc. Natl. Acad. Sci. U.S.A. 100, 1541–1546. doi: 10.1073/pnas.0337542100, PMID: 12563033 PMC149868

[B20] Hanahan D. (1983). Studies on transformation of *Escherichia coli* with plasmids. J. Mol. Biol. 166, 557–580. doi: 10.1016/s0022-2836(83)80284-8, PMID: 6345791

[B21] Hawkins P. Morton D. B. Burman O. Dennison N. Honess P. Jennings M.. (2011). A guide to defining and implementing protocols for the welfare assessment of laboratory animals: eleventh report of the BVAAWF/FRAME/RSPCA/UFAW Joint Working Group on Refinement. Lab. Anim. 45, 1–13. doi: 10.1258/la.2010.010031, PMID: 21123303

[B22] Hernández-García M. Barbero-Herranz R. Bastón-Paz N. Díez-Aguilar M. López-Collazo E. Márquez-Garrido F. J.. (2024). Unravelling the mechanisms causing murepavadin resistance in *Pseudomonas aeruginosa*: lipopolysaccharide alterations and its consequences. Front. Cell Infect. Microbiol. 14, 1446626. doi: 10.3389/fcimb.2024.1446626, PMID: 39711784 PMC11659217

[B23] Hittle L. E. Powell D. A. Jones J. W. Tofigh M. Goodlett D. R. Moskowitz S. M.. (2015). Site-specific activity of the acyltransferases HtrB1 and HtrB2 in *Pseudomonas aeruginosa* lipid A biosynthesis. Pathog. Dis. 73, ftv053. doi: 10.1093/femspd/ftv053, PMID: 26223882 PMC4626592

[B24] Huang T. W. Lam I. Chang H. Y. Tsai S. F. Palsson B. O. Charusanti P. (2014). Capsule deletion via a lambda-Red knockout system perturbs biofilm formation and fimbriae expression in *Klebsiella pneumoniae* MGH 78578. BMC Res. Notes 7, 13. doi: 10.1186/1756-0500-7-13, PMID: 24398052 PMC3892127

[B25] Huszczynski S. M. Hao Y. Lam J. S. Khursigara C. M. (2020). Identification of the *Pseudomonas aeruginosa* O17 and O15 O-specific antigen biosynthesis loci reveals an ABC transporter-dependent synthesis pathway and mechanisms of genetic diversity. J. Bacteriol. 202, e00347-20. doi: 10.1128/JB.00347-20, PMID: 32690555 PMC7484189

[B26] Jesudason T. (2024). WHO publishes updated list of bacterial priority pathogens. Lancet Microbe 5, 100940. doi: 10.1016/j.lanmic.2024.07.003, PMID: 39079540

[B27] Kawasaki K. Ernst R. K. Miller S. I. (2004). 3-O-deacylation of lipid A by PagL, a PhoP/PhoQ-regulated deacylase of *Salmonella* Typhimurium, modulates signaling through Toll-like receptor 4. J. Biol. Chem. 279, 20044–20048. doi: 10.1074/jbc.M401275200, PMID: 15014080

[B28] Kawasaki K. Teramoto M. Tatsui R. Amamoto S. (2012). Lipid A 3'-O-deacylation by *Salmonella* outer membrane enzyme LpxR modulates the ability of lipid A to stimulate Toll-like receptor 4. Biochem. Biophys. Res. Commun. 428, 343–347. doi: 10.1016/j.bbrc.2012.10.054, PMID: 23085233

[B29] Killough M. Rodgers A. M. Ingram R. J. (2022). *Pseudomonas aeruginosa*: recent advances in vaccine development. Vaccines (Basel). 10, 1100. doi: 10.3390/vaccines10071100, PMID: 35891262 PMC9320790

[B30] Knirel Y. A. Bystrova O. V. Kocharova N. A. Zahringer U. Pier G. B. (2006). Conserved and variable structural features in the lipopolysaccharide of *Pseudomonas aeruginosa* . J. Endotoxin Res. 12, 324–336. doi: 10.1179/096805106X118906, PMID: 17254386

[B31] Lee D. G. Urbach J. M. Wu G. Liberati N. T. Feinbaum R. L. Miyata S.. (2006). Genomic analysis reveals that *Pseudomonas aeruginosa* virulence is combinatorial. Genome Biol. 7, R90. doi: 10.1186/gb-2006-7-10-r90, PMID: 17038190 PMC1794565

[B32] Livak K. J. Schmittgen T. D. (2001). Analysis of relative gene expression data using real-time quantitative PCR and the 2(-Delta Delta C(T)) Method. Methods 25, 402–408. doi: 10.1006/meth.2001.1262, PMID: 11846609

[B33] Lofblom J. Kronqvist N. Uhlen M. Stahl S. Wernerus H. (2007). Optimization of electroporation-mediated transformation: *Staphylococcus carnosus* as model organism. J. Appl. Microbiol. 102, 736–747. doi: 10.1111/j.1365-2672.2006.03127.x, PMID: 17309623

[B34] Maeshima N. Fernandez R. C. (2013). Recognition of lipid A variants by the TLR4-MD-2 receptor complex. Front. Cell Infect. Microbiol. 3, 3. doi: 10.3389/fcimb.2013.00003, PMID: 23408095 PMC3569842

[B35] Mata-Haro V. Cekic C. Martin M. Chilton P. M. Casella C. R. Mitchell T. C. (2007). The vaccine adjuvant monophosphoryl lipid A as a TRIF-biased agonist of TLR4. Science 316, 1628–1632. doi: 10.1126/science.1138963, PMID: 17569868

[B36] McClelland M. Sanderson K. E. Spieth J. Clifton S. W. Latreille P. Courtney L.. (2001). Complete genome sequence of *Salmonella enterica* serovar Typhimurium LT2. Nature 413, 852–856. doi: 10.1038/35101614, PMID: 11677609

[B37] Morton D. B. Griffiths P. H. (1985). Guidelines on the recognition of pain, distress and discomfort in experimental animals and an hypothesis for assessment. Vet. Rec 116, 431–436. doi: 10.1136/vr.116.16.431, PMID: 3923690

[B38] Moskowitz S. M. Ernst R. K. Miller S. I. (2004). PmrAB, a two-component regulatory system of *Pseudomonas aeruginosa* that modulates resistance to cationic antimicrobial peptides and addition of aminoarabinose to lipid A. J. Bacteriol 186, 575–579. doi: 10.1128/JB.186.2.575-579.2004, PMID: 14702327 PMC305751

[B39] Mulani M. S. Kamble E. E. Kumkar S. N. Tawre M. S. Pardesi K. R. (2019). Emerging strategies to combat ESKAPE pathogens in the era of antimicrobial resistance: A review. Front. Microbiol. 10, 539. doi: 10.3389/fmicb.2019.00539, PMID: 30988669 PMC6452778

[B40] Needham B. D. Carroll S. M. Giles D. K. Georgiou G. Whiteley M. Trent M. S. (2013). Modulating the innate immune response by combinatorial engineering of endotoxin. Proc. Natl. Acad. Sci. U.S.A. 110, 1464–1469. doi: 10.1073/pnas.1218080110, PMID: 23297218 PMC3557076

[B41] Nowicki E. M. O'Brien J. P. Brodbelt J. S. Trent M. S. (2015). Extracellular zinc induces phosphoethanolamine addition to *Pseudomonas aeruginosa* lipid A via the ColRS two-component system. Mol. Microbiol. 97, 166–178. doi: 10.1111/mmi.13018, PMID: 25846400 PMC4715879

[B42] Park B. S. Song D. H. Kim H. M. Choi B. S. Lee H. Lee J. O. (2009). The structural basis of lipopolysaccharide recognition by the TLR4-MD-2 complex. Nature 458, 1191–1195. doi: 10.1038/nature07830, PMID: 19252480

[B43] Pérez-Ortega J. Van Harten R. M. Van Boxtel R. Plisnier M. Louckx M. Ingels D.. (2021). Reduction of endotoxicity in *Bordetella bronchiseptica* by lipid A engineering: Characterization of *lpxL1* and *pagP* mutants. Virulence 12, 1452–1468. doi: 10.1080/21505594.2021.1929037, PMID: 34053396 PMC8168481

[B44] Reynolds C. M. Ribeiro A. A. McGrath S. C. Cotter R. J. Raetz C. R. H. Trent M. S. (2006). An outer membrane enzyme encoded by *Salmonella* Typhimurium *lpxR* that removes the 3'-acyloxyacyl moiety of lipid A. J. Biol. Chem. 281, 21974–21987. doi: 10.1074/jbc.M603527200, PMID: 16704973 PMC2702521

[B45] Rutten L. Geurtsen J. Lambert W. Smolenaers J. J. Bonvin A. M. de Haan A.. (2006). Crystal structure and catalytic mechanism of the LPS 3-O-deacylase PagL from *Pseudomonas aeruginosa* . Proc. Natl. Acad. Sci. U.S.A. 103, 7071–7076. doi: 10.1073/pnas.0509392103, PMID: 16632613 PMC1564273

[B46] Santamarina-Fernández R. Fuentes-Valverde V. Silva-Rodríguez A. García P. Moscoso M. Bou G. (2025). *Pseudomonas aeruginosa* vaccine development: lessons, challenges, and future innovations. Int. J. Mol. Sci. 26, 2012. doi: 10.3390/ijms26052012, PMID: 40076637 PMC11900337

[B47] Sen-Kilic E. Blackwood C. B. Huckaby A. B. Horspool A. M. Weaver K. L. Malkowski A. C.. (2021). Defining the Mechanistic Correlates of Protection Conferred by Whole-Cell Vaccination against *Pseudomonas aeruginosa* Acute Murine Pneumonia. Infect. Immun. 89, e00451-20. doi: 10.1128/IAI.00451-20, PMID: 33199354 PMC7822147

[B48] Sieuwerts S. de Bok F. A. Mols E. de vos W. M. Vlieg J. E. (2008). A simple and fast method for determining colony forming units. Lett. Appl. Microbiol. 47, 275–278. doi: 10.1111/j.1472-765X.2008.02417.x, PMID: 18778376

[B49] Simpson B. W. Trent M. S. (2019). Pushing the envelope: LPS modifications and their consequences. Nat. Rev. Microbiol. 17, 403–416. doi: 10.1038/s41579-019-0201-x, PMID: 31142822 PMC6913091

[B50] Stover C. K. Pham X. Q. Erwin A. L. Mizoguchi S. D. Warrener P. Hickey M. J.. (2000). Complete genome sequence of *Pseudomonas aeruginosa* PAO1, an opportunistic pathogen. Nature 406, 959–964. doi: 10.1038/35023079, PMID: 10984043

[B51] Thaipisuttikul I. Hittle L. E. Chandra R. Zangari D. Dixon C. L. Garrett T. A.. (2014). A divergent *Pseudomonas aeruginosa* palmitoyltransferase essential for cystic fibrosis-specific lipid A. Mol. Microbiol. 91, 158–174. doi: 10.1111/mmi.12451, PMID: 24283944 PMC3935289

[B52] Zhang Y. F. Han K. Chandler C. E. Tjaden B. Ernst R. K. Lory S. (2017). Probing the sRNA regulatory landscape of *P. aeruginosa*: post-transcriptional control of determinants of pathogenicity and antibiotic susceptibility. Mol. Microbiol. 106, 919–937. doi: 10.1111/mmi.13857, PMID: 28976035 PMC5738928

