# Supplementary material for: LpxR and PagL expression in live attenuated auxotrophic Pseudomonas aeruginosa vaccines modulates lipid A reactogenicity in vitro while preserving immunogenicity
Source: Front Cell Infect Microbiol. 2025 Oct 16;15:1664169. doi: 10.3389/fcimb.2025.1664169 (PMC12571757; doi:10.3389/fcimb.2025.1664169)
Supplement: Supplementary file 1 [file Table1.docx]

Supplementary Material

# LpxR and PagL expression in live attenuated auxotrophic *Pseudomonas aeruginosa* vaccines modulates lipid A reactogenicity *in vitro* while preserving immunogenicity

**Table S1.** Dose (in CFU) used to inoculate BALB/c mice with PAO1 Δ*murI*, PAO1 Δ*murI* p*lpxR*_ST_, and PAO1 Δ*murI* p*pagL* vaccine strains via different administration routes. Mean values ± SD of doses per mouse are indicated.

| Administration routes |  | PAO1 Δ*murI* (CFU) | PAO1 Δ*murI* p*lpxR*_ST_ (CFU) | PAO1 Δ*murI* p*pagL* (CFU) |
| --- | --- | --- | --- | --- |
| Intranasal |  | 1.3 ± 0.42 × 10^8^ | 8.6 ± 4.81 × 10^7^ | 6.2 ± 1.7 × 10^7^ |
| Intranasal plus intramuscular | IN | 6.9 ± 0.28 × 10^7^ | 1.3 ± 0.52 × 10^7^ | 2.1 ± 1.39 × 10^7^ |
|  | IM | 5.9 ± 2.31 × 10^7^ | 1.3 ± 1.16 × 10^7^ | 5.7 ± 4.80 × 10^7^ |
| Intradermal |  | 1.7 ± 1.24 × 10^7^ | 3.4 ± 4.41 × 10^7^ | 2.9 ± 4.16 × 10^7^ |
| Subcutaneous |  | 9.5 ± 4.53 × 10^7^ | 9.3 ± 7.7 × 10^7^ | 1.4 ± 1.46 × 10^8^ |
